# Molecular Aspect of Good Eating Quality Formation in *Japonica* Rice

**DOI:** 10.1371/journal.pone.0018385

**Published:** 2011-04-06

**Authors:** Ming-Mao Sun, Sailila E. Abdula, Hye-Jung Lee, Young-Chan Cho, Long-Zhi Han, Hee-Jong Koh, Yong-Gu Cho

**Affiliations:** 1 Department of Crop Science, Chungbuk National University, Cheongju, Korea; 2 National Institute of Crop Science, Rural Development Administration, Suwon, Korea; 3 Institute of Crop Science, Chinese Academy of Agricultural Sciences, Beijing, China; 4 Department of Plant Science, Seoul National University, Seoul, Korea; University College London, United Kingdom

## Abstract

The composition of amylopectin is the determinant of rice eating quality under certain threshold of protein content and the ratio of amylose and amylopectin. In molecular biology level, the fine structure of amylopectin is determined by relative activities of starch branching enzyme (SBE), granule-bound starch synthase (GBSS), and soluble starch synthase (SSS) in rice grain under the same ADP-Glucose level. But the underlying mechanism of eating quality in molecular biology level remains unclear. This paper reports the differences on major parameters such as SNP and insertion-deletion sites, RNA expressions, and enzyme activities associated with eating quality of *japonica* varieties. Eight *japonica* rice varieties with significant differences in various eating quality parameters such as palatability and protein content were used in this experiment. Association analysis between nucleotide polymorphism and eating quality showed that S12 and S13 loci in *SBE1*, S55 in *SSS1*, S58 in *SSS2A* were significantly associated with apparent amylose content, alkali digestion value, setback viscosity, consistency viscosity, pasting temperature, which explained most of the variation in apparent amylose content, setback viscosity, and consistency viscosity; and explained almost all variations in alkali digestion value and pasting temperature. Thirty-five SNPs and insertion-deletions from *SBE1*, *SBE3*, *GBSS1*, *SSS1*, and *SSS2A* differentiated high or intermediate palatability rice varieties from low palatability rice varieties. Correlation analysis between enzyme activities and eating quality properties revealed that SBE25 and SSS15/W15 were positively correlated with palatability, whereas GBSS10 and GBSS15 were negatively correlated. Gene expressions showed that *SBE1* and *SBE3* expressions in high palatability varieties tended to be higher than middle and low palatability varieties. Collectively, *SBE1*, *SBE3*, *SSS1*, and *SSS2A*, especially *SBE1* and *SBE3* could improve eating quality, but *GBSS1* decreased eating quality. The results indicated the possibility of developing high palatability cultivars through modification of key genes related to *japonica* rice eating quality formation in starch biosynthesis.

## Introduction

Rice (*Oryza sativa* L.) is the staple food for more than half of the world's population. Each year many new rice varieties are being developed and released with high yield, and high resistance to biotic and abiotic stresses, but their eating quality remains to be improved. Eating quality has now become the primary consideration of final rice-eating consumers and breeding programs. Cultivars with better eating quality are also required for food industry and rice seed producers. Although *indica* rice varieties are widely planted worldwide, consumers in Northeast Asia preferred *japonica* rice. Also, *japonica* rice is becoming more and more popular with worldwide consumers due to its moderate elasticity and stickiness [1]–[3].

Polished rice is mainly made up of starch, protein, lipid, and moisture. The protein content of polished rice in 22 *japonica* rice varieties ranged from 5.9 to 7.9% [3], and 6.0–13.6% in brown rice among 1,518 Chinese *japonica* varieties [4]. Starch comprises 76.7–78.4% in polished rice with 14% moisture content [5]. Eating quality of rice is thus mainly influenced by starch property. Moreover, eating quality of rice is a very complex trait, and the palatability from Toyo taste meter is significantly and positively correlated with palatability from sensory test [3]. Therefore, palatability is directly related to rice eating quality. In physicochemical level, apparent amylose content [6], gel consistency [7], gelatinization temperature [8] or alkali digestion value, pasting properties [9], chain length distribution of amylopectin [10], and protein content [11] are thought to be important parameters affecting eating quality. Rice eating quality is partially affected by environmental factors such as growing temperature and soil fertility, but mainly determined by genetic control [12].

**Figure 1:**
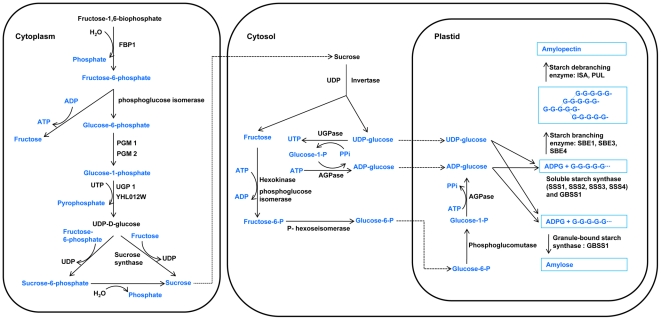
Main pathway of starch biosynthesis including sucrose synthesis, sucrose degradation and starch synthesis in rice. FBP1, fructose-1,6-bisphophatase1; PGM, phosphoglucomutase; AGPase, ADP-glucose pryophosphorylase; PPi, pyrophosphate.

In molecular biological aspects, sucrose derived from glucose and fructose is synthesized in cytoplasm, and then transported into cytosol in which sucrose is degraded by invertase. Using degraded products of sucrose, fructose and UDP-glucose, starch is synthesized by multiple subunits or isoforms of five classes of enzymes: ADP-glucose pyrophosphorylase (AGP), soluble starch synthase (SSS), granule-bound starch synthase (GBSS), starch branching enzyme (SBE), and starch debranching enzyme (DBE) [13]–[15] (Figure 1). Moreover, GBSS is not only responsible for amylose synthesis, but also involved in amylopectin synthesis, especially in forming the extra-long chains of amylopectin [16]. Among the five enzymes, SBE, GBSS, SSS, and DBE are contributing to the fine structure of amylopectin [17]. SBE in rice grain contains SBE1, SBE3 (QEIIa, or BEIIb), and SBE4 (QEIIb, or BEIIa) isoforms [18]–[20]. GBSS has two isoforms, GBSS1 in rice grain and GBSS2 in rice leaf [21]. SSS contains SSS1, SSS2A (SSSII-3), SSS2B (SSSII-2), SSS2C (SSSII-1), SSS3A (SSSIII-2), SSS3B (SSSIII-1), and SSS4A (SSSIV-1), SSS4B (SSSIV-2) [22]. DBE contains isoamylase (ISA) and pullulanase (PUL) in rice. ISA has at least three isoforms, ISA1, ISA2, and ISA3, and only one PUL [23].

The enzymes and genes involved in fine structure of amylopectin play distinct roles, but jointly comprise of the genetic dissection of rice eating quality. Using 70 rice varieties, association analysis between 18 genes related to starch synthesis and amylose content, gel consistency, gelatinization temperature showed that *Waxy* (*GBSS1*) and *ALK* (*SSS2A*) are central in determining rice eating and cooking quality by affecting amylose content, gel consistency and gelatinization temperature [24]. *Waxy* functions as the sole major gene for both amylose content and gel consistency but as a minor gene affecting gelatinization temperature. *ALK* is the sole major gene controlling gelatinization temperature but as a minor gene affecting amylose content and gel consistency. The two genes affect two properties simultaneously: both *ISA* and *SBE3* affect gel consistency and gelatinization temperature. Furthermore, several minor genes are specific for each property: *SSS3A*, *AGPlar* (AGP large subunit), *PUL*, and *SSS1* for amylose content, *AGPiso* (AGP large subunit isoform) for gel consistency, and *SSS4B* for gelatinization temperature. The correlations among amylose content, gel consistency, and gelatinization temperature were caused by the joint action of these associated genes.

In the analysis of sequence variation, Glu-88 (gag) and Gly-604 (ggc) in *SSS2A* of *indica* cultivars IR36 and Kasalath were replaced by Asp-88 (gac) and Ser-604 (agc), respectively, in both *japonica* cultivars Nipponbare and Kinmaze *SSS2A* whereas Val-737 (gtg) and Leu-781 (ctc) in *indica SSS2A* were replaced by Met-737 (atg) in Nipponbare and Phe-781 (ttc) in Kinmaze *SSS2A*. These results confirmed that the SSS2A activity determines the type of amylopectin structure of rice starch to be either the typical *indica* type or *japonica* type, by playing a specific role in the synthesis of the long B_1_ chains by elongating short A and B_1_ chains, notwithstanding the presence of functional two additional *SSS2* genes, a single *SSS1* gene, two *SSS3* genes, and two *SSS4* genes in rice plants [25]. Moreover, 25 single nucleotide polymorphism (SNP) and insertion-deletion sites in *SSS2A* were found in 30 rice varieties and GC/TT polymorphism could differentiate rice varieties with high or intermediate GT from those with low GT at a rate of about 90% correct prediction for 509 varieties [26].

Activities of AGP at all rice grain filling stages and activities of SBE at the early and middle grain filling stages were not correlated with rice eating quality components including gel consistency, alkali spreading value, and amylose content. SSS activity at the early grain filling stage was negatively correlated with gel consistency and alkali spreading value, and positively correlated with amylose content. Activities of SSS at middle and late grain filling stages and SBE at the late grain filling stage were positively correlated with gel consistency and alkali spreading value, and negatively correlated with amylose content [27]. The SSS activity at whole grain filling stages was not correlated with palatability, but the activities of AGP and SBE at 12, 24, and 30 days after heading were correlated with palatability. Moreover, the activities of AGP, SSS, and SBE were correlated with Rapid Visco Analyzer (RVA) pasting properties [28]. Changes in nucleotide sequences and mRNA expressions of genes involved in SBE, GBSS, and SSS, and activities of SBE, GBSS, and SSS in rice grain, and the relationship between enzyme activities of SBE and SSS and rice eating quality are studied little. There is no report on the correlations between nucleotide sequence variation and rice palatability, between mRNA expression and rice palatability, between GBSS activity and rice palatability.

In this study, our aim was to find the relationships between nucleotide sequence variations of *SBE1, SBE3, GBSS1, SSS1*, and *SSS2A* and rice eating quality, between activities of SBE, GBSS1, and SSS at different grain filling stages and rice eating quality, between gene expressions of *SBE1* and *SBE3* at different grain filling stages and rice eating quality, and to explore the molecular biology aspect of good eating quality formation in *japonica* rice which provide the basic ideas for breeding good eating quality *japonica* rice.

## Results

### Eating quality traits

Palatability from Toyo taste meter served as an important and direct index in estimating eating quality of *japonica* rice instead of sensory test [3], [29]. The physicochemical properties of eight *japonica* rice varieties were summarized in Table 1 and Table S3.

**Table 1:** Means and ranges of eating quality parameters in eight *japonica* rice varieties.

Parameter a	Mean ± SD	Pr>F	Range	CV (%) b	Skewness	Kurtosis
P	60.4±7.28	<0.0001	49.7–70.4	12.04	−0.23	−1.19
AAC (%)	17.47±1.31	<0.0001	14.35–18.67	7.49	−1.53	1.90
PC (%)	7.09±1.00	<0.0001	5.78–8.54	14.12	0.22	−1.44
ADV	5.8±1.65	<0.0001	1.5–6.5	28.62	−2.25	3.09
PV *	282.70±24.69	<0.0001	241.45–317.56	8.74	−0.05	−0.77
HPV *	174.60±15.24	0.0003	158.79–203.68	8.73	0.73	−0.56
CPV *	280.17±18.74	0.0001	247.37–307.57	6.69	−0.32	−0.49
BDV *	108.10±23.65	<0.0001	75.60–148.65	21.87	0.05	−0.68
SBV *	-2.53±32.04	<0.0001	−67.60 to 39.71	1268.70	−0.64	0.14
CTV *	105.58±10.63	<0.0001	81.05–115.31	10.07	−1.48	1.24
PT (°C)	68.84±2.12	<0.0001	68.00–74.28	3.08	2.27	3.14

a P, palatability value; AAC, apparent amylose content; PC, protein content; ADV, alkali digestion value; PV, peak value; HPV, hot pate viscosity; CPV, cool paste viscosity; BDV, breakdown value; SBV, setback viscosity; CTV, consistency viscosity; PT, pasting temperature;

b CV, coefficient of variation; * RVU: rapid visco unit.

**Figure 2:**
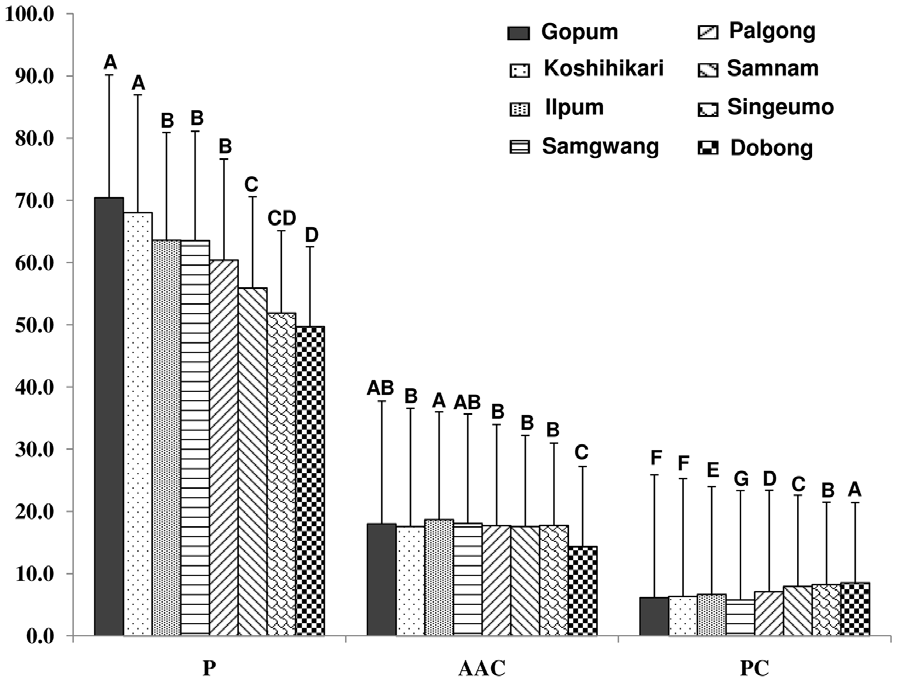
Palatability (P), apparent amylose content (AAC) (%), and protein content (PC) (%) in polished rice of eight *japonica* varieties. Letters A–F, difference is significant at 0.05 level.

All traits showed significant differences among varieties and exhibited a wide range of variation, especially palatability (P) with 49.7–70.4, apparent amylose content (AAC) of 14.35–18.67, and protein content (PC) of 5.78–8.54 (Figure 2). The palatability was higher in Gopum and Koshihikari followed by Ilpum and Palgong. Lowest palatability was in Samnam, Singeumo, and Dodong. The protein content in high palatability varieties was significantly lower than low palatability varieties. There was no evident trend in apparent amylose content of high and low palatability varieties. Moreover, palatability value could be divided into three groups: high (Gopum and Koshihikari), middle (Ilpum, Samgwang, and Palgong), and low (Samnam, Singeumo, and Dobong). Among middle palatability group, Palgong was relatively lower than Ilpum and Samgwang (Figure 2).

Correlation analysis revealed that palatability was negatively correlated with protein content (Table 2). However, no significant correlation was observed to the other traits including apparent amylose content, alkali digestion value (ADV), and RVA pasting properties indicating that the palatability is a complex trait in which many factors are involved, but mainly affected by protein content. Apparent amylose content and alkali digestion value were significantly correlated with RVA pasting properties such as breakdown viscosity, setback viscosity, consistency viscosity, and pasting temperature.

**Table 2:** Correlation matrix of rice eating quality parameters.

Parameter	AAC	P	PC	ADV	PV	HPV	CPV	BDV	SBV	CTV
P †	0.63									
PC	−0.64	−0.93**								
ADV	0.97**	0.59	−0.58							
PV	−0.58	0.12	−0.09	−0.53						
HPV	0.19	0.36	−0.21	0.21	0.36					
CPV	0.68	0.53	−0.46	0.70	−0.10	0.82*				
BDV	−0.72*	−0.10	0.04	−0.68	0.81*	−0.25	−0.62			
SBV	0.83**	0.21	−0.20	0.81*	−0.82*	0.19	0.65	−0.97**		
CTV	0.92**	0.41	−0.51	0.93**	−0.68	0.02	0.59	−0.72*	0.86**	
PT	−0.96**	−0.58	0.55	−1.00**	0.51	−0.23	−0.70	0.67	−0.79*	−0.91**

† P, palatability value; AAC, apparent amylose content; PC, protein content; ADV, alkali digestion value; PV, peak value; HPV, hot pate viscosity; CPV, cool paste viscosity; BDV, breakdown value; SBV, setback viscosity; CTV, consistency viscosity; PT, pasting temperature;

**, significant at 0.01; *, at 0.05 level.

### Nucleotide sequence differences

Five genes related to fine structure formation of amylopectin were selected to identify the differences in the association analysis of eating quality. To identify base pair differences in genomic regions, the polymerase chain reaction (PCR) products of these genes were sequenced as follows: 8,756 bp for *SBE1*, 10,287 bp for *SBE3*, 3,444 bp for *GBSS1*, 6,914 bp for *SSS1*, and 2,314 bp for *SSS2A* (Table S1). Sequenced fragments were detected in 60 SNP/insertion-deletion (InDel) sites for 5 genes among eight *japonica* varieties. The polymorphic sites were listed in Table S4. Of the 60 polymorphic sites, 17 were located in coding regions, and the diversity of coding sequences was much lower than non-coding sequences throughout the genes investigated.

For *SBE1* gene, the S6, S8, S12, S13, S16, S18 and S19 sites “AGGGTG-”were the same in Gopum, Koshihikari, Ilpum, and Samgwang, but they were partially replaced by “GAAACAT” in Palgong, Samnam, Singeumo, and Dobong. The S1, S10, S15, and S17 sites “CAT-” were the same in Palgong, Samnam, Singeumo, and Dobong, but they were partially replaced by “TGGG/A” in Gopum, Koshihikari, Ilpum, and Samgwang. For *SBE3* gene, the S23, S25–S29, S36, S42, S43, S45, S46 sites “CATGTTAAGCC” were same in Palgong, Samnam, Singeumo, and Dobong, but they were partially replaced by “ACCTG-TG–A” in Gopum, Koshihikari, Ilpum, and Samgwang. Though we detected sequences of *GBSS1*, *SSS1*, and *SSS2A* in some SNP/insertion-deletion sites, we did not find strong evidence as to differences related to eating quality compared to *SBE1* and *SBE3* in all *japonica* rice varieties. It indicated that the “AGGGTTG-” linkage sites may be advantageous for high eating quality formation, but the “CATGTTAAGCC” linkage sites may be disadvantageous for high eating quality formation in *japonica* rice varieties.

Association analysis between eating quality properties and SNPs/insertion-deletions showed that S12 and S13 loci in *SBE1*, S55 in *SSS1*, S58 in *SSS2A* were significantly associated with apparent amylose content, alkali digestion value, setback viscosity (SBV), consistency viscosity (CTV), and pasting temperature (PT), which explained most of the variation in apparent amylose content, setback viscosity, and consistency viscosity, and almost all variations in alkali digestion value and pasting temperature (*R^2^* = 0.99) (Table 3). S40 in *SBE3*, and S48 and S49 in *GBSS1* were significantly associated with peak viscosity (PV) and consistency viscosity at P<0.05, which explained about 60% of total variation. S3, S4, and S5 in *SBE1* were significantly associated with breakdown viscosity (BDV), indicating that the breakdown viscosity was partially controlled by *SBE1*. However, palatability was not significantly associated with any single SNP or insertion-deletion locus detected.

**Table 3:** Highly associated SNPs and insertion-deletions with eating quality properties identified under general linear model.

Trait	Locus a	*R^2^* b	F value	P value	Trait	Locus	*R^2^*	F value	P value
AAC †	S12	0.92	73.75	1.4×10^−4^	BDV	S4	0.54	7.18	3.7×10^−2^
	S13	0.92	73.75	1.4×10^−4^		S5	0.53	6.67	4.2×10^−2^
	S55	0.92	73.75	1.4×10^−4^	SBV	S12	0.63	10.29	1.8×10^−2^
	S58	0.92	73.75	1.4×10^−4^		S13	0.63	10.29	1.8×10^−2^
ADV	S12	0.99	1605.56	1.6×10^−8^		S55	0.63	10.29	1.8×10^−2^
	S13	0.99	1605.56	1.6×10^−8^		S58	0.63	10.29	1.8×10^−2^
	S55	0.99	1605.56	1.6×10^−8^	CTV	S12	0.83	28.93	1.7×10^−3^
	S58	0.99	1605.56	1.6×10^−8^		S13	0.83	28.93	1.7×10^−3^
PV	S40	0.67	12.16	1.3×10^−2^		S55	0.83	28.93	1.7×10^−3^
	S48	0.67	12.16	1.3×10^−2^		S58	0.83	28.93	1.7×10^−3^
	S49	0.67	12.16	1.3×10^−2^		S40	0.55	7.39	3.5×10^−2^
	S53	0.57	7.94	3.0×10^−2^		S48	0.55	7.39	3.5×10^−2^
	S5	0.57	7.89	3.0×10^−2^		S49	0.55	7.39	3.5×10^−2^
HPV	S22	0.59	8.64	2.6×10^−2^		S14	0.52	6.58	4.3×10^−2^
	S36	0.59	8.64	2.6×10^−2^	PT	S12	0.99	10928.62	5.2×10^−11^
	S59	0.59	8.64	2.6×10^−2^		S13	0.99	10928.62	5.2×10^−11^
	S17	0.75	7.53	3.1×10^−2^		S55	0.99	10928.62	5.2×10^−11^
CPV	S51	0.58	8.12	2.9×10^−2^		S58	0.99	10928.62	5.2×10^−11^
BDV	S3	0.54	7.18	3.7×10^−2^					

† AAC, apparent amylose content; ADV, alkali digestion value; PV, peak value; HPV, hot pate viscosity; CPV, cool paste viscosity; BDV, breakdown value; SBV, setback viscosity; CTV, consistency viscosity; PT, pasting temperature;

a SNP and insertion-deletion (see Table S4);

b explained variation.

**Figure 3:**
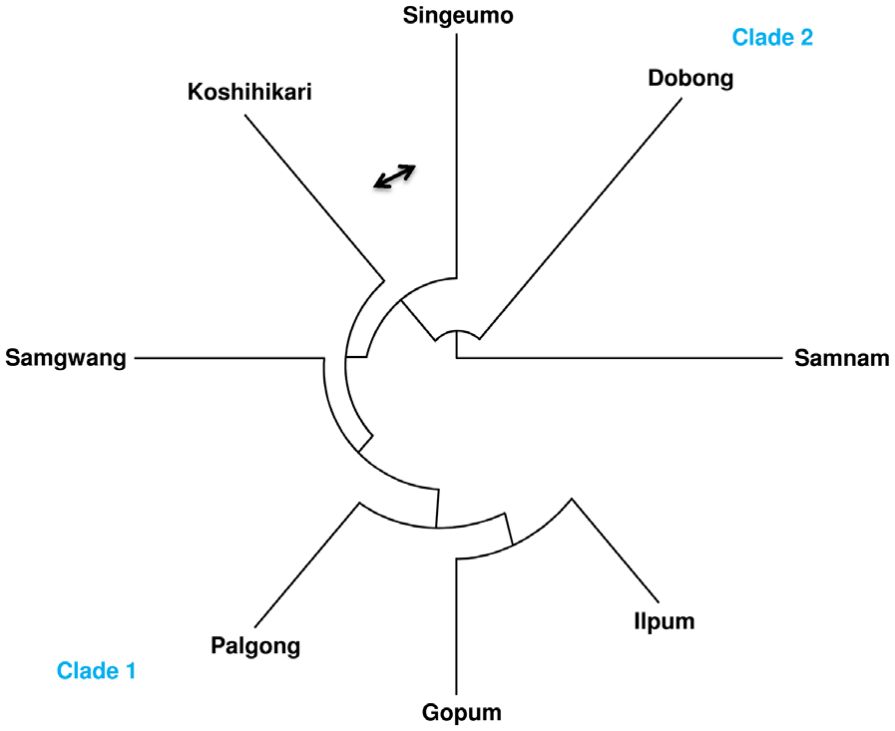
UPGMA tree based on 35 SNPs and insertion-deletions from five genes in eight *japonica* varieties. The 35 SNPs and insertion-deletions are as follows: S2, S3, S4, S5, S6, S7, S8, S9, S11, S12, S13, S14, S16, S18, S19, S20, S21, S22, S24, S30, S31, S32, S33, S34, S35, S37, S38, S39, S40, S41, S44, S48, S55, S58, S60 (See Table S4).

Phylogenetic analysis was performed using 35 SNPs and insertion-deletions filtered from *SBE1*, *SBE3*, *GBSS1*, *SSS1*, and *SSS2A* (Figure 3). In UPGMA tree, high and intermediate palatability varieties were placed in Clade1, and low palatability varieties were placed in Clade2, indicating that palatability was controlled by a few polymorphic sites together at the genomic level resulted in enzymatic differences. These results showed that the five genes would be very important to eating quality formation.

### Enzyme activities

**Figure 4:**
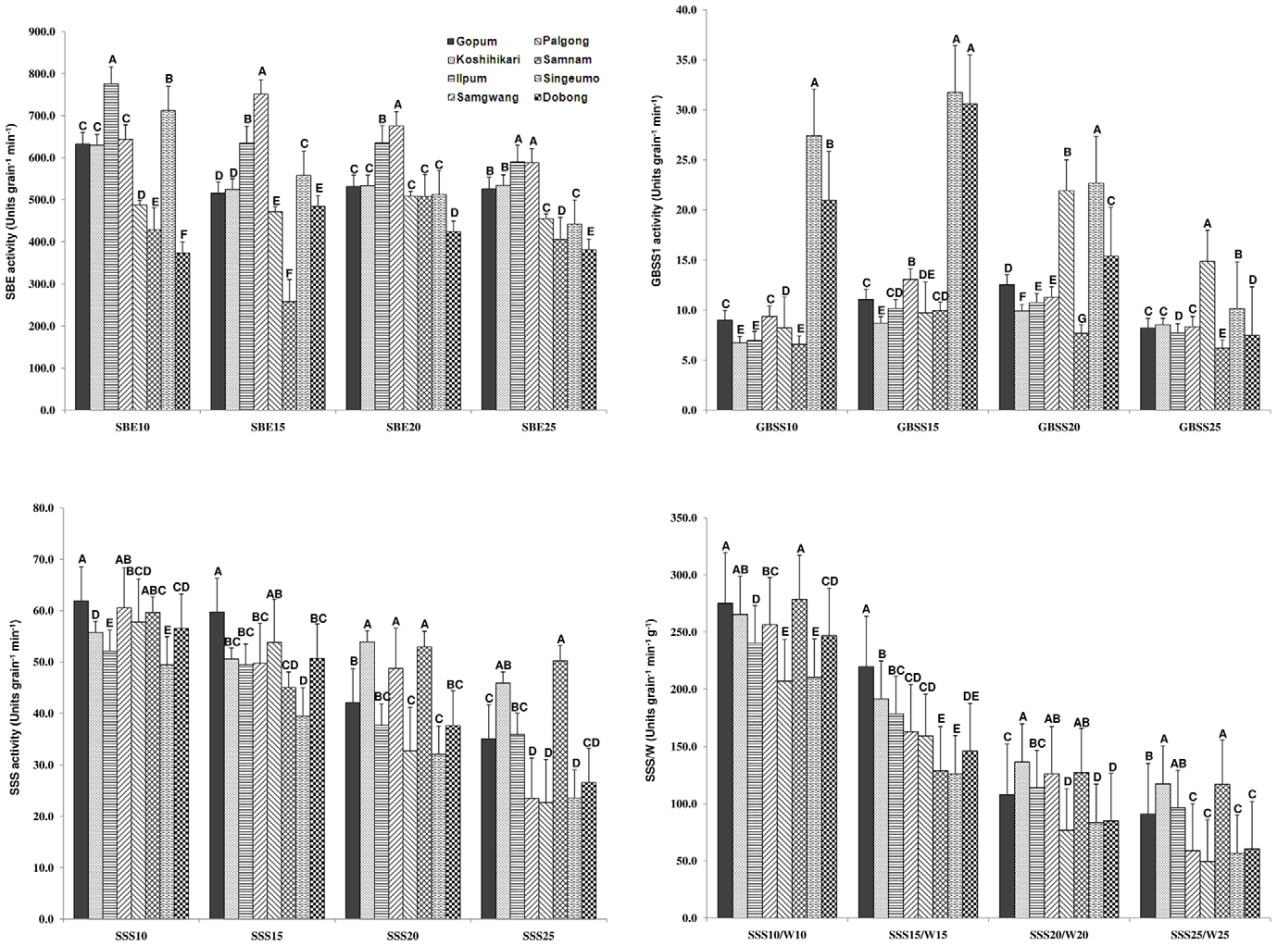
Enzyme activities related to amylopectin biosynthesis during rice grain filling in eight *japonica* varieties. Letters A–G indicate significant differences at 0.05 level.

The enzyme activities of SBE, GBSS1, SSS, and the ratio of SSS activity to grain weight (SSS/W) in developing rice grains were presented in Figure 4. Eight varieties showed different enzyme activities with diverse maximum peak times during grain filling stage.

For starch branching enzyme, the peak values of enzyme activity were formed during 10–15 days after flowering in six varieties except for Palgong and Samnam. The activities of SBE at 25 days after flowering were higher in Gopum, Koshihikari, Ilpum, and Samgwang than those in Palgong, Samnam, Singeumo, and Dobong. For GBSS1, the peak values of enzyme activity were formed during 15–20 days after flowering. For soluble starch synthase, the peak values of enzyme activity were showed at 10 days after flowering. The differences were existed among eight varieties, but there were no evident differences for SSS activities between high and inferior palatability groups. In GBSS1, Singeumo and Dobong showed much higher activities than the other varieties during grain filling, but unexpectedly high during 10–15 days after flowering. Also, Palgong was high during 20–25 days after flowering. This implies that the high enzyme activity of GBSS1 was reflected to low palatability based on the negative correlation between GBSS1 activity and palatability (Table 4).

**Table 4:** Correlation analysis between eating quality traits and enzyme activities during rice grain filling.

Trait	Enzyme	Correlation coefficient
AAC †	SBE10 a	0.74*
	SBE20	0.74*
P	SBE25	0.79*
	GBSS10	−0.73*
	GBSS15	−0.79*
	SSS15/W15	0.90**
BDV	SBE10	−0.71*
SBV	SBE10	0.76*
CTV	SBE10	0.73*

† AAC, apparent amylose content; P, palatability value; BDV, breakdown value; SBV, setback viscosity; CTV, consistency viscosity;

a SBE10, starch branching enzyme activity at 10 days after flowering; W15, grain weight at 15 days after flowering;

**,*: correlation is significant at 0.01 and 0.05 level, respectively.

Correlation analyses revealed that SBE25, GBSS10, GBSS15, and SSS15/W15 were significantly correlated with palatability (Table 4). Among them, SBE25 was positively correlated with palatability, suggesting that the SBE activity at late grain filling stage played an important role in good eating quality formation. However, the activities of GBSS10 and GBSS15 were negatively correlated with palatability and resulted in low eating quality. The correlation between SSS activity and palatability was not significant (data not shown), therefore SSS activity/grain weight was employed to detect the correlation (Table 4). The SSS15/W15 was positively correlated with palatability, implying that SSS activity/grain weight at middle stage was reflected to form the high palatability of rice grain. SBE activities were positively correlated with apparent amylose content at 10 and 20 days after flowering. Partial apparent amylose content was from long strains of increased amylopectin, and resulted in high apparent amylose content within certain threshold. Moreover, SBE activity at 10 days after flowering was positively correlated with consistency viscosity and setback viscosity, but negatively correlated with breakdown viscosity. This result was consistent with report of Shen et al. [28], implying that SBE played an important role in rice amylopectin composition. These results indicated that SBE and SSS/W could be controlled by modifying gene expression to improve rice eating quality, but GBSS1 reduced eating quality during rice grain filling stage.

### mRNA expressions

**Figure 5:**
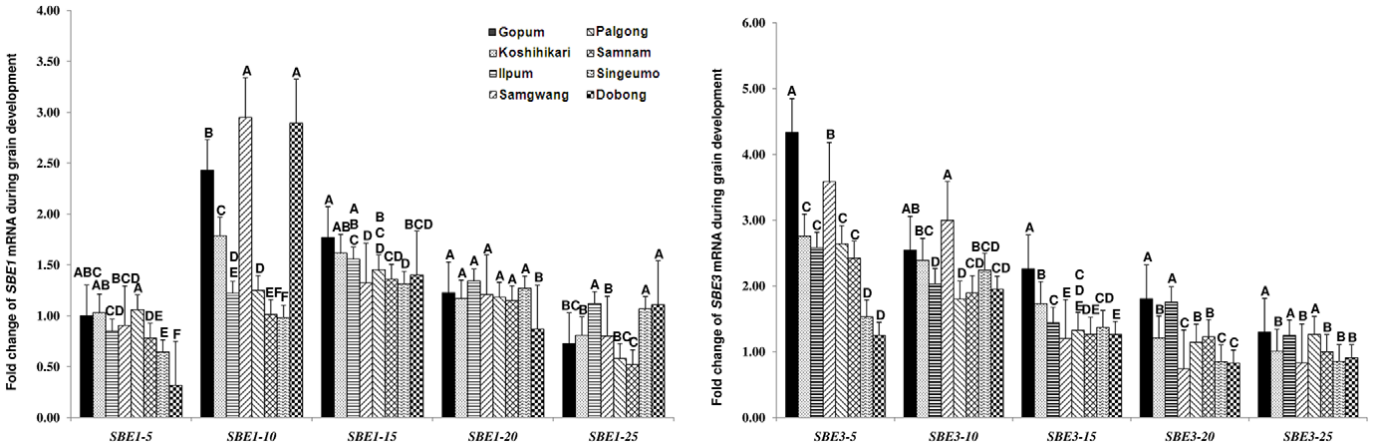
*SBE1* and *SBE3* expressions based on real-time RT-PCR during grain filling in eight *japonica* varieties. Letters A–F indicates difference is significant at 0.05 level.

Since starch branching enzyme was important determinant of eating quality by association analysis, real –time RT-PCR of SBE1 and SBE3 was carried out to elucidate the effect of gene expression to eating quality (see the primer information in Table S2). Using eight *japonica* varieties, the mRNA expressions of *SBE1* and *SBE3* tended to increase gradually to peak values, thereafter descended during late grain filling period (Figure 5). The peak values of *SBE1* expression formed during 10–15 days after flowering, and the peak values of *SBE3* formed during 5–10 days after flowering, which were consistent with report of Ohdan et al. [22]. According to palatability, the eight *japonica* varieties were divided into three groups: high (Gopum, Koshihikari), middle (Ilpum, Samgwang, Palgong), and low (Samnam, Singeumo, Dobong) palatability. T-tests showed that there were significant differences among high, middle, and low palatability groups. Alkali digestion value and cool paste viscosity were significantly different between middle and low.

The differences of *SBE1* and *SBE3* expressions at 5, 10, 15, 20, and 25 days after flowering among high, middle, and low palatability groups were detected using t-tests (LSD), respectively (Table 5). *SBE1* mRNA expression at 5 days after flowering (*SBE1-5*) and *SBE3-5* expressions were significantly different between high and low, and between middle and low groups. *SBE1-15* and *SBE3-15* expressions were significantly different between high and middle groups, and between high and low groups. *SBE3-20* expression was significantly different between high and low groups. *SBE3-25* expression was significantly different between high and low groups, and between middle and low groups at P<0.05.

**Table 5:** mRNA expressions of *SBE1* and *SBE3* related to eating quality properties among high, middle, and low palatability groups.

Parameter	High P group (2) a	Middle P group (3)	Low P group (3)	H-M	H-L	M-L
P †	69.2	62.5	52.5	**	**	**
ADV	6.3	6.5	4.7	ns	ns	*
CPV	288.73	288.03	266.61	ns	ns	*
*SBE1-5*	1.02	0.94	0.58	ns	**	**
*SBE1-15*	1.62	1.44	1.36	**	**	ns
*SBE3-5*	3.54	2.94	1.73	ns	**	**
*SBE3-15*	2.00	1.32	1.30	**	**	ns
*SBE3-20*	1.51	1.21	0.97	ns	**	ns
*SBE3-25*	1.15	1.11	0.92	ns	*	*

† P, palatability value; ADV, alkali digestion value; CPV, cool paste viscosity; *SBE1-5*, *SBE1* mRNA expression at 5 days after flowering;

a , number in the parenthesis means the number of varieties;

**,*: difference is significant at 0.01 and 0.05 level, respectively; ns, not significant.

Correlation analysis between eating quality properties and mRNA expressions of *SBE1* and *SBE3* involving *SBE1-15* and *SBE3-15* played an important role in palatability difference between high and middle groups. Interestingly, mRNA expressions of *SBE1-5*, *SBE1-15*, *SBE3-5*, *SBE3-15*, *SBE3-20*, and *SBE3-25* contributed a lot to the palatability variation between high and low groups. Among them, *SBE1-5*, *SBE3-5*, and *SBE3-25* were important in palatability differentiation between middle and low groups. These results suggested that mRNA expressions of *SBE1* and *SBE3* were important to form good eating quality in *japonica* varieties, especially *SBE1* and *SBE3* expressions at both 5 and 15 days after flowering. *SBE1-5*, *SBE3-5*, and *SBE3-25* also played an important role in alkali digestion value and cool paste viscosity variations between middle and low palatability groups.

## Discussion

Rice (*Oryza sativa* L.) is the most important staple food of East Asia and the most densely populated landscapes of China, Korea, Japan, Southeast Asia, and South Asia [30], which contains two subspecies, *japonica* and *indica*. Molecular and archaeobotanical evidences have shown that *japonica* and *indica* rice originated from distinct gene pools in the wild ancestor *Oryza rufipogon* [31]. Consumers in northeastern Asian countries and regions such as Korea, northern China, Taiwan, and Japan prefer the gelatinous to sticky spectrum of *japonica* rice [2], [3], [32]. The preference for eating glutinous endosperm starch or sticky foods had been established fairly early in the evolution of cereal farming. This particular preference for eating glutinous foods maybe the effective basis for the later selection of sticky endosperm mutants from normal non-glutinous cereal crops such as rice, foxtail millet, common millet, barley, and maize [32]. This indicated the existence of rice eating quality preference which exerted selective pressure in favor of sticky endosperm form of rice in Northeast Asia [33].

The palatability from Toyo taste meter is positively and significantly correlated with palatability from sensory test, and thus the palatability from Toyo taste meter is considered an important and direct index in the evaluation of rice eating quality [3], [29]. There was no correlation between palatability and any RVA pasting properties, between palatability and amylose content, between palatability and alkali digestion value, indicating that apparent amylose content, alkali digestion value, and RVA pasting properties could not be a good indicator for eating quality evaluation of *japonica* rice. The protein content which showed negative correlation was the only trait to affect the palatability as previously reported [3], [34]. Setback viscosity in 23 rice varieties ranged from −80 to 132, and the coefficient of variation was 275.47 [35]. Lestari et al. [3] reported that setback viscosity in 22 *japonica* rice varieties ranged from −94.75 to 25.00, and the coefficient of variation was 318.71. In this study, setback viscosity ranged from −67.60 to 39.71, and the coefficient of variation was 1268.70 which was produced by small number of varieties. The alkali digestion value of Dobong was much smaller than the other varieties. On the contrary, the pasting temperature of Dobong was much higher than the other varieties (Table S3).

Nucleotide sequence composition is the basis of gene expression and gene function, and the SNP and insertion-deletion sites of genes related to amylopectin structure among different rice varieties have been reported. The G/T polymorphism at 5′ splice site of *GBSS1* 1^st^ intron affects the rice apparent amylose content. Cultivars with 18% or less amylose content have the sequence, AGTTATA at the 5′ splice site, while cultivars with a higher proportion of amylose have AGGTATA [36]. All eight *japonica* rice varieties have the sequence AGTTATA at the 5′ splice site. 24 SNPs and 1 insertion-deletion in the *SSS2A* gene among 30 rice varieties were detected, the low GT rice varieties had the TT allele, whereas the high GT varieties had the GC allele at sites 4,329/4,330 [26]. The C/A, C/T, C/T, G/A, and G/T SNPs at 2,739, 2,781, 2,920, 2,955, and 3,003 sites, respectively, did not show the polymorphism in the eight *japonica* varieties, displaying C, C, C, G, and T alleles, respectively. The ACTAGT/ACTACT SNP at the 3′UTR of *SBE3*, and CGTG/CGCG SNP at the 14^th^ intron of *SBE1*, and insertion-deletion of *Tourist-Os*6 transposon at 335 bp upstream of *SBE1* start codon accounted for ∼70% of the observed variations of hot and cool viscosities, and for 40% of the observed variation of peak viscosity and consistency [37]. In the association analysis between microsatellite alleles of genes related to starch synthesis and eating quality parameters with 270 recombinant inbred lines, *GBSS1* was associated with apparent amylose content, peak viscosity, hot paste viscosity, cool paste viscosity, breakdown viscosity, setback viscosity, pasting temperature, hardness, adhesiveness, and cohesiveness; *SSS1* was associated with apparent amylose content, hot paste viscosity, cool paste viscosity, breakdown viscosity, setback viscosity, hardness, adhesiveness, and cohesiveness; the association between *SBE1* and eating quality parameters were not significant [38].

60 SNP and insertion-deletion sites were detected for *SBE1*, *SBE3*, *GBSS1*, *SSS1*, and *SSS2A* nucleotide sequences in eight *japonica* varieties. The SNP and insertion-deletion sites were not even in allele distribution among eight *japonica* varieties. It indicated that single SNP or insertion-deletion might not be a good measure to evaluate rice eating quality, and thus several SNP and insertion-deletion sites should be combined. Apparent amylose content, alkali digestion value, setback viscosity, and pasting temperature were associated with *SBE1*, *SSS1*, and *SSS2A*. Peak viscosity was associated with *SBE1*, *SBE3*, *GBSS1*, and *SSS1*. Hot paste viscosity was associated with *SBE1*, *SBE3*, and *SSS2A*. Cool paste viscosity was associated with *GBSS1*. Breakdown viscosity was associated with *SBE1*. Consistency viscosity was associated with *SBE1*, *SBE3*, *GBSS1*, *SSS1*, and *SSS2A*. 35 SNPs and insertion-deletions from *SBE1*, *SBE3*, *GBSS1*, *SSS1*, and *SSS2A* jointly differentiated the low palatability and high or middle palatability *japonica* groups. Tian et al. [24] reported the apparent amylose content was controlled by *GBSS1*, *SSS2A*, and *SSS1*, whereas gelatinization temperature was controlled by *SSS2A*, *GBSS1*, *ISA*, *SBE3*, and *SSS4B* in 33 *indica* and 37 *japonica* rice varieties. Apparent amylose content, hot paste viscosity, breakdown viscosity, cool paste viscosity, consistency viscosity, setback viscosity, and pasting temperature were controlled by *GBSS1* in 88 *indica* rice varieties, whereas apparent amylose content, cool paste viscosity, and consistency viscosity were controlled by *AGPL1-4*, hot paste viscosity was controlled by *SBE3*, setback viscosity and consistency viscosity were controlled by *GBSS1* in 41 *japonica* rice varieties [39].

The peak values of starch branching enzyme activity appeared during 10–15 days after flowering in Gopum, Koshihikari, Ilpum, Samgwang, Singeumo, and Dobong, whereas Palgong and Samnam appeared at 20 days after flowering, which were consistent with previous reports [28], [40]–[42]. The peak values of GBSS 1 activity in eight *japonica* varieties appeared during 15–20 days after flowering, which were in good agreement with the report of Liu et al. [43]. The peak values of SSS activity in all *japonica* varieties appeared at 10 days after flowering, and then descended gradually during rice grain filling stage, which were consistent with previous reports [41]–[43].

SBE25 and SSS15/W15 were positively correlated with palatability, whereas GBSS10 and GBSS15 were negatively correlated with palatability. The results indicated that SBE and SSS/W could improve rice eating quality, but GBSS1 decreased the eating quality. Shen et al. [28] reported that palatability was negatively correlated with SBE activity at 12 days after heading, and positively correlated with SBE activities at 24 and 30 days after heading, respectively, but no correlation between SSS activity and palatability was detected.

The peak values of *SBE1* expression formed during 10–15 days after flowering, and the peak values of *SBE3* formed during 5–10 days after flowering were consistent with the report of Ohdan et al. [22]. mRNA expression is located in an intermediate process between initial nucleotide diversity and final enzyme activity, which is important in rice eating quality formation.

We divided eight *japonica* varieties into three groups according to palatability. The differences in eating quality properties, *SBE1* and *SBE3* expressions between high and middle, between high and low, between middle and low palatability groups were detected. This method was used to compare the gene expressions between *indica* and *japonica* variety groups, and found the relationships between cooking and eating quality properties and gene expressions [39]. Therefore, this method is feasible in gene expression analysis. *SBE1-15* and *SBE3-15* expressions in high palatability group were higher than those in middle palatability group. *SBE1-5*, *SBE1-15*, *SBE3-5*, *SBE3-15*, *SBE3-20*, and *SBE3-25* expressions in high palatability group were higher than those in low palatability group. *SBE1-5*, *SBE3-5*, and *SBE3-25* expressions in middle palatability group were higher than those in low palatability group, indicating that *SBE1* and *SBE3* expressions tended to be higher in high palatability varieties compared to middle and low palatability varieties. The *SBE1* and *SBE3* were important to good eating quality formation in *japonica* varieties.

## Materials and Methods

### Plant Materials and Field Trials

The field experiment was conducted in the rice field of Chungbuk National University in 2009. Eight *japonica* rice varieties, Koshihikari, Gopum, Ilpum, Samgwang, Palgong, Samnam, Singeumo, and Dobong, with different eating quality were used. All varieties were developed in Korea except Koshihikari which was developed in Japan.

The varieties were sown in a seedling-growing tray and placed in the greenhouse for 3 weeks in May, 2009. Grown seedlings were transplanted in the experimental farm spaced at 30×15 cm with one seedling per hill and arranged in randomized complete block design replicated three times. The cultivation and management were performed according to the rice cultivation standards of the experimental farm in Chungbuk National University.

The young leaf tissues of eight varieties were collected at the tillering stage for genomic DNA extraction. Thirty uniformly flowered panicles were chosen and recorded the flowering date for each variety in each plot. Six tagged panicles from each variety in each plot were sampled at 5, 10, 15, 20, and 25 days after flowering. Collected samples were immediately frozen in liquid nitrogen for 5 min, and then stored at −80°C for enzyme activity and mRNA expression analyses. Matured rice seeds were harvested separately in each plot, and air-dried for 2 weeks before measuring the palatability and physicochemical properties.

### DNA Extraction and Purification

Genomic DNA was extracted in extraction buffer (100 mM Tris-HCl pH 8.0, 500 mM NaCl, 50 mM EDTA, 1.25% SDS, Sodium bisulfate 0.38 g/100 ml) as described by Cho et al. [44] with some modifications. 0.2 g of ground powder of fresh rice leaf tissue was placed in 2 ml microcentrifuge tube, and 900 µl of 65°C preheated extraction buffer (pH 7.8–8.0) was added. After incubation in water bath at 65°C for 30 min, 700 µl of phenol: chloroform: isoamyl alcohol (25∶24∶1) was added into the tube and mixed for 10 min, and centrifugation was carried out at 13,000 rpm for 10 min. The upper phase was transferred into a new 2 ml tube and equal volume of chloroform: isoamyl alcohol (24∶1) was added and mixed for 10 min. The mixed samples were centrifuged at 13,000 rpm for 10 min and the upper phase was transferred into a new 1.5 ml tube containing 4 µl of 10 mg/ml RNase1. After incubation at 37°C for 30 min, 0.7 volume of precooled isopropanol was added and mixed well, and centrifuged at 13,000 rpm at 4°C for 15 min. The supernatant was discarded and 1 ml of 70% ethanol was applied to wash the DNA pellet and centrifuged at 13,000 rpm for 5 min. DNA pellet was air-dried and suspended in 50 µl of double-distilled water.

### Primer Design, PCR Reaction, and Sequencing

The nucleotide sequences of *SBE1* (LOC_Os06g51084), *SBE3* (LOC_Os02g32660), *GBSS1* (LOC_Os06g04200), *SSS1* (LOC_Os06g06560), and *SSS2A* (LOC_Os06g12450) were obtained by searching the Gramene database (http://www.gramene.org). Using genomic DNA sequences, primers were designed with Primer3 online software (http://biotools.umassmed.edu/bioapps/primer3_www.cgi), and primer analysis was carried out using Oligo 6 software. The primers used for sequencing were listed in Table S1. Polymerase chain reaction (PCR) was carried out in a 50 µl reaction mixture containing 80 ng of template DNA, 5 µl of 10×buffer, 4 µl of dNTP mixture (2.5 mM each), 1.25 units of Ex Taq polymerase (Takara), and 1.25 µl each of forward and reverse primers (10 µM). The PCR reaction was performed at 94°C for 5 min as a hot start, followed by 25 cycles of 94°C for 30 sec, 55°C for 30 sec and 72°C for 30 sec. Moreover, the annealing temperature and extension time were adjusted according to different primer pairs and expected PCR product sizes. The final extension was followed by one cycle of 10 min at 72°C. The reactions were carried out in PTC-100 programmable thermal controller (MJ Research Inc.). Amplified PCR products were analyzed by electrophoresis on 1% agarose gel stained with ethidium bromide, and purified by PCR DNA purification kit (GeneAll). After that, the purified PCR products were sequenced at COSMO Genetech Company, and the sequences reported in this paper have been deposited in the GenBank database (accession nos. HQ712126-HQ712165). DNA fragments sequenced of 8 rice varieties were analyzed with clustalw of DDBJ (http://clustalw.ddbj.nig.ac.jp/).

### RNA Extraction and First-Strand Synthesis of cDNA

Total RNAs were isolated from rice grains using the RNeasy mini kit (Qiagen) according to the manufacturer's instruction with some modifications. 100 mg of frozen ground seed powder was placed into cold 1.5 ml tube, and then 600 µl of RLC buffer was added. After immediately rapid and strong vortex, centrifugation was carried out at 13,000 rpm for 2 min. The supernatant was rapidly transferred into lilac spin column, and then centrifuged at 12,000 rpm for 2 min. 280 µl of 100% ethanol was added into the flowthrough, mixed 4–5 times by pipetting, and then the sample was transferred into pink RNeasy spin column. After centrifugation at 10,000 rpm for 1 min, 700 µl of RW1 buffer was added into the pink spin column. Centrifugation was carried out at 10,000 rpm for 1 min, and then 500 µl of RPE buffer was used to wash RNA twice at 10,000 rpm for 1 min. After centrifugation for 2 min at 10,000 rpm, 50 µl of RNase-free water was added into the pink spin column in 1.5 ml tube, and centrifuged again at 10,000 rpm for 1 min. The RNA concentration was detected in Nanodrop ND-1000 spectrophotometer (NanoDrop Technologies, Inc. USA), and stored at −80°C freezer. Total RNAs were cleaned using DNase 1 kit (Cat. No: 18068-015, Invitrogen), and the first-strand cDNA synthesis was performed by reverse transcription of mRNA using Oligo(dT)_20_ primer and SuperScript™ III Reverse Transcriptase (Cat. No: 18080-051, Invitrogen).

### Quantitative Real-Time RT-PCR

The deduced amino acid sequences of *SBE1* and *SBE3* were blasted at NCBI/BlAST website, and the clustalw (DDBJ/Clustalw site) of amino acid sequences from 15 species was carried out, and conserved domains of these amino acid sequences were searched. Using cDNA sequences responsible for the conserved domains of proteins, primers were designed with Primer3 online software, and primer analysis was carried out using Oligo 6 software. Quantitative real-time RT-PCR was performed on the DNA Engine Opticon 2 instrument (MJ Research, Waltham, MA) in 20 µl reaction volume containing SYBR Green, a fluorophore which binds to all double-strand DNA (F-410L, qPCR kit from FINNZYMES, Finland). The primers used for real-time RT-PCR were shown in Table S2. PCR conditions were as follows: 8 min at 95°C followed by 42 cycles of 33 sec at 94°C, 32 sec at 55°C, 22 sec at 72°C, and final extension of 10 min at 72°C. Finally, the relative quantification of gene expression was analyzed using the 2-ddCt method [45] by normalization to internal control *Actin-1*.

### SBE Activity Assay

Enzyme activity of starch branching enzyme including SBE1, SBE3, and SBE4 together in rice grains was assayed as described by Yamanouchi and Nakamura [18] with some modifications. Fifteen frozen rice endosperms were weighed and homogenized in 4 ml of cold extraction buffer (50 mM HEPES-NaOH pH 7.4, 4 mM MgCl_2_, 50 mM 2-mercaptoethanol, 12.5% (v/v) glycerol) contained in a precooled mortar with a pestle on ice. The homogenate was transferred into a precooled 40 ml tube, and 6 ml of extraction buffer was added for two times to clean the mortar. 40 ml tubes were centrifuged at 15,000 rpm at 2°C for 20 min, and supernatants were filtered through a filter paper (0.45 µm, Whatman, Whatman International LTD). The filtrates were used for enzyme preparations.

Starch branching enzyme was assayed by monitoring stimulation of α-glucan synthesis from glucose-1-P by rabbit muscle phosphorylase a [46]–[47]. 100 µl of enzyme preparation was added into 100 µl of cold reaction buffer (50 mM HEPES-NaOH pH 7.0, 50 mM glucose-1-P, 2.5 mM AMP, 1.2 units of phosphorylase a from rabbit muscle), and was incubated at 30°C for 30 min. The reaction was terminated by adding 50 µl 1 M HCl, and mixed with 500 µl of dimethylsulfoxide. 700 µl of fresh iodine-potassium iodide solution (2 g KI +200 ml distilled water +0.2 g I_2_) was added into the solution, and then incubated at 30°C for 30 min in the dark. The SBE activity was determined by measuring the absorbance at 540 nm in UV spectrophotometer (Shimadzu). The controls were prepared as follows: 100 µl of enzyme preparation was added into 50 µl 1 M HCl, mixed well, and 100 µl of cold reaction buffer was added into the mixture. The other steps were same as reaction samples. The blank solution was made using 100 µl of cold extraction buffer to replace 100 µl enzyme preparation and the other steps were same as reaction samples. Three replications were carried out in this experiment.

The starch branching enzyme activity was calculated as follows: SBE activity  = ΔA×100×V_R_×V_T_/(T×N×d×V_S_×V_S_); ΔA, absorbance of samples at 30 min minus absorbance of samples at 0 min; T, time (minute); N, grain number; d, light path in cm; 100, 0.01 optical density as 1 unit which has similar function as ε or %E; V_R_, reaction volume (enzyme sample volume + working reagent volume + stop solution volume) (ml); V_S_, enzyme sample volume (ml); V_T_, total enzyme sample volume (ml). So SBE activity = ΔA×100×1.45×10/(30×15×1×0.1×0.1) = 322.22×ΔA (Units grain^−1^ min^−1^).

### GBSS1 and SSS Extraction

GBSS1 and SSS enzyme extractions were conducted as described by Umemoto and Terashima [48] with some modifications. Fifteen frozen rice endosperms were weighed and hand-homogenized in 2 ml of ice-cold extraction buffer (100 mM Tris pH 7.2, 2 mM EDTA, 2 mM DTT, and 10% (v/v) ethanediol in a precooled mortar with a pestle on ice. The homogenate was transferred into a precooled 40 ml tube, and 1 ml of extraction buffer was used to clean the mortar, and then transferred to 40 ml tube. The homogenate was then centrifuged at 15,000 rpm and 2°C for 20 min. The supernatant was collected and filtered through a membrane filter (0.45 µm, Whatman, Whatman International LTD), and the filtrate was used for the assay of SSS. The pellet was washed three times by suspension and centrifugation using extraction buffer. The washed pellet was resuspended in 3 ml extraction buffer and used for GBSS1 assay.

### GBSS1 Activity Assay

The activity of GBSS1 was assayed as described by Nakamura et al. [47] with some modifications. 0.15 ml of GBSS1 enzyme preparation was added into 0.15 ml of solution A (50 mM HEPES-NaOH pH 7.4, 1.6 mM ADP glucose, 0.7 mg amylopectin, 15 mM DTT), and incubated at 30°C for 1 hour (mixed every 10 min). The enzyme was inactivated by placing the mixture in a boiling-water bath for 30 s, and incubated on ice for 10 min. The mixture was then added by 0.1 ml of solution B (50 mM HEPES-NaOH pH 7.4, 4 mM PEP, 200 mM KCl, 10 mM MgCl_2_, and 1.5 units of pyruvate kinase), and incubated at 30°C for 30 min. The ADP produced by the starch synthase reaction was conversed to ATP and the resulting solution was heated in a boiling-water bath for 30 s, and incubated on ice for 10 min, and then subjected to centrifugation at 15,000 rpm and 2°C for 15 min. The supernatant (0.3 ml) was mixed with 0.3 ml of solution C (50 mM HEPES-NaOH pH 7.4, 10 mM glucose, 20 mM MgCl_2_, 2 mM NADP). The GBSS1 activity was measured as the increase in absorbance of 340 nm after the addition of 1 µl each of hexokinase (1.4 units) and G-6-P dehydrogenase (0.35 units) at 25°C for 30 min.

The controls were prepared as follows: 0.15 ml of GBSS1 enzyme preparation was inactivated in a boiling-water bath for 30 s, and incubated on ice for 10 min, and then solution A was added. The other steps were same as reaction samples. The blank solution was made using 0.15 ml of cold extraction buffer to replace 0.15 ml GBSS1 preparation, and the other steps were same as reaction samples. Three replications were carried out in this experiment.

The enzyme activity of granule-bound starch synthase 1 was calculated as follows: GBSS1 activity = ΔA×100×V_R_×V_T_/(T×N×d×V_S_×V_S_); ΔA, absorbance of samples at 30 min minus absorbance of samples at 0 min; T, time (minute); N, grain number; d, light path in cm; 100, 0.01 optical density as 1 unit which has similar function as ε or %E; V_R_, reaction volume (enzyme sample volume + working reagent volume + stop solution volume) (ml); V_S_, enzyme sample volume (ml); V_T_, total enzyme sample volume (ml). So GBSS1 activity = ΔA×100×0.602×3/(60×15×0.15×3/4×0.15×3/4) = 15.86×ΔA (Units grain^−1^ min^−1^).

### SSS Activity Assay

The enzyme activity of SSS was assayed as described by Nishi et al. [49] with some modifications. 0.15 ml of SSS preparation was added into 0.15 ml of buffer A (0.5 M citrate-Na pH 7.5, 50 mM Bicine-NaOH pH 7.5, 1.7 mM ADP-Glucose, 0.7 mg oyster glycogen (Type II, Sigma), 16.7 mM DTT), and incubated at 30°C for 20 min. The enzyme was inactivated by placing the mixture in a boiling-water bath for 30 s, and incubated on ice for 10 min. The mixture was supplemented with 0.1 ml of Buffer B (50 mM HEPES-NaOH pH 7.4, 10 mM phosphocreatine, 200 mM KCl, 10 mM MgCl_2_, and 10 µl of 5 mg ml^−1^ creatine phosphokinase (5 units, TypeI, Sigma)), and then incubated at 30°C for 30 min. The ADP produced by the soluble starch synthase reaction was conversed to ATP, and the resulting solution was heated in a boiling-water bath for 30 s, and incubated on ice for 10 min, and then subjected to centrifugation at 15,000 rpm and 2°C for 15 min. The supernatant (0.3 ml) was mixed with 0.3 ml of buffer C (125 mM HEPES-NaOH pH 7.4, 10 mM glucose, 20 mM MgCl_2_, and 150 µg NADP). The SSS activity was measured as the increase in absorbance of 340 nm after the addition of 1.1 µl of hexokinase (1.4 units/µl, Roche Diagnostics, Tokyo) and 1.5 µl of G-6-P dehydrogenase (0.35 units/µl, Type XV, Sigma) at 25°C for 30 min.

The controls were prepared as follows: 0.15 ml of soluble starch synthase preparation was inactivated in a boiling-water bath for 30 s, and incubated on ice for 10 min, and then buffer A was added. The other steps were same as reaction samples. The blank solution was made using 0.15 ml of cold extraction buffer to replace 0.15 ml of soluble starch synthase preparation, and the other steps were same as reaction samples. Three replications were carried out in this experiment.

The enzyme activity of soluble starch synthase was calculated as follows: SSS activity = ΔA×100×V_R_×V_T_/(T×N×d×V_S_×V_S_); ΔA, absorbance of samples at 30 min minus absorbance of samples at 0 min; T, time (minute); N, grain number; d, light path in cm; 100, 0.01 optical density as 1 unit which has similar function as ε or %E; V_R_, reaction volume (enzyme sample volume + working reagent volume + stop solution volume) (ml); V_S_, enzyme sample volume (ml); V_T_, total enzyme sample volume (ml). So SSS activity = ΔA×100×0.6026×3/(20×15×0.15×3/4×0.15×3/4) = 47.61×ΔA (Units grain^−1^ min^−1^). SSS10/W10, SSS15/W15, SSS20/W20, and SSS25/W25 were calculated by SSS activities at 10, 15, 20, and 25 days after flowering were divided by 15 grain weight at 10, 15, 20, and 25 days after flowering, respectively.

### Eating Quality Traits Evaluation

For palatability and physicochemical analyses, rice grains were dehulled and milled to 91% yield. The analyses were performed as described by Lestari et al. [3] with some modifications. Palatability was detected by a rice taste measuring system (Toyo taste meter, model MA-90) according to the manufacture's instruction (TRCM Co.) (Toyo Rice Polishing Machine Factory, Japan). Protein content was determined by the micro-kjeldahl method [50] and calculated with the determined value using total nitrogen multiplied by the conversion coefficient 5.95. The amylose content of milled rice was measured using the relative absorbance of starch-iodine color in a digested solution of 100-mesh rice flour as described by Perez and Juliano [51]. The amylose content measured from iodine-binding capacity was named as apparent amylose content. Thus, actual amylose content was derived from apparent content by subtracting the contribution of the long chains of amylopectin [52]. The alkali digestion value was estimated using the alkali digestion test and spreading scores [8].

RVA pasting properties were detected with a Rapid Visco Analyzer (RVA) according to the manufacture's instruction (NewPort Sci. Co., Australia). Rice starch paste profile was described by seven parameters. They were as follows: peak viscosity (PV), through viscosity or hot paste viscosity (HPV), final viscosity or cool paste viscosity (CPV), breakdown viscosity (BDV = PV-HPV), setback viscosity (SBV = CPV-PV), consistency viscosity (CTV = CPV-HPV), and pasting temperature as described by Bao and Xia [53] and Shen et al. [28]. All the viscosity parameters were expressed in rapid visco units.

### Statistical Analysis

Analysis of variance was performed using SAS version 9.1(SAS Institute Inc., Cary, NC, USA). Significant differences were determined using t-tests (LSD) for comparison of means. Correlation analyses among rice eating quality parameters and enzyme activities were conducted using Statistix version 8.0 (http://www.statistix.com/). The general linear model (GLM) was used in the analysis of associations between nucleotide polymorphisms and various rice eating quality parameters, which was performed with Tassel version 2.1 (http://www.maizegenetics.net/tassel). Moreover, phylogenetic analysis was also performed using Tassel 2.1 software.

## Supporting Information

Table S1 **Primers used in the sequencing of genes related to rice amylopectin biosynthesis.**
(DOC)

Table S2 **Primers for** ***SBE1*** **and** ***SBE3*** **expressions in developing rice grains.**
(DOC)

Table S3 **Eating quality parameters for eight** ***japonica*** **rice varieties.**
(DOC)

Table S4 **Polymorphic sites of genes related to rice amylopectin biosynthesis of eight** ***japonica*** **varieties.**
(DOC)
