# Supplementary material for: Molecular Aspect of Good Eating Quality Formation in Japonica Rice
Source: PLoS One. 2011 Apr 6;6(4):e18385. doi: 10.1371/journal.pone.0018385 (PMC3071818; doi:10.1371/journal.pone.0018385)
Supplement: Table S1 — Primers used in the sequencing of genes related to rice amylopectin biosynthesis. (DOC) [file pone.0018385.s001.doc]

**Table S1. Primers used in the sequencing of genes related to rice amylopectin biosynthesis.**

| **Gene** | **Primer** | **Forward** | **Primer sequences** | **Reverse** | **Primer sequences** |
| --- | --- | --- | --- | --- | --- |
| *SBE1* | SBE1-1 | SBE1-F1 | GCAAGCAACCTCATTTGGAT | SBE1-R1 | GTGAAATCCTACCGGCAAGT |
|  | SBE1-2 | SBE1-F2 | TGCTGTGTCTCACCTCCTCTT | SBE1-R2 | GGCATTGCATGATGTCCAAG |
|  | SBE1-3 | SBE1-F3 | TCTGCAGTGTTGTTCTTTTGTG | SBE1-R3 | AAGGAACCTGCTTACTGTCAAT |
|  | SBE1-4 | SBE1-F4 | CCTCCTCCCCTTTTCTTCTG | SBE1-R4 | TCCTGATCTGCTGCTGACTG |
|  | SBE1-5 | SBE1-F5 | CCTATGCCGAGAGCCATGAT | SBE1-R5 | CCACCAAAGACCAAAGCATC |
|  | SBE1-6 | SBE1-F6 | GCATTGCACTCCAAAAGGTT | SBE1-R6 | ACATGCCGTTCCGATTTATG |
|  | SBE1-7 | SBE1-F7 | CTTTGAACGTGGAGATTTGG | SBE1-R 7 | TCTGAACGCCAAAGTGTCAT |
|  | SBE1-8 | SBE1-F8 | TATCTCTGGTGGCTGGAAGG | SBE1-R8 | TCCCTCTCACCAATCACCAC |
| *SBE3* | SBE3-1 | SBE3-F1 | GGAGGTTGGAGCTGAAGTTG | SBE3-R1 | CACCCAGAGAAGTGATGTGC |
|  | SBE3-2 | SBE3-F2 | TGAAGTCAATATGCCCATGCT | SBE3-R2 | ACGGCTAATTGTTGACGAATG |
|  | SBE3-3 | SBE3-F3 | GGTGGGGTTCTCAACTTAGC | SBE3-R3 | GGTAGGCATGGTGTTATTCG |
|  | SBE3-4 | SBE3-F4 | ACCTTTGCTGGATGGTTGATT | SBE3-R4 | TTGTCCACCAGAAAGCCAAC |
|  | SBE3-5 | SBE3-F5 | TAGCCCTCATGTTGGTGAAC | SBE3-R5 | TACTCCTCGAGCCACCATCT |
|  | SBE3-6 | SBE3-F6 | TTTAGCTCATTGGCCTCTGG | SBE3-R6 | CATCAAATGAGCAGCCCTTC |
|  | SBE3-7 | SBE3-F7 | TGCCACTAGCTCCGCTATCT | SBE3-R7 | TGGAACTCACACGACCACTG |
|  | SBE3-8 | SBE3-F8 | TGGCTGTTCCTGACAAATGG | SBE3-R8 | ACTGTCCACATAGCCCATGC |
|  | SBE3-9 | SBE3-F9 | GCCCATGCTCATCGTATCTC | SBE3-R9 | CTCACCAGGATGTCCGAACT |
|  | SBE3-10 | SBE3-F10 | GGTGAAATGACGAGCTGCAC | SBE3-R10 | GCAAGGAATGAGCACAACAG |
| *GBSS1* | GBSS1-1 | GBSS1-F1 | GGTTGGAAGCATCACGAGTT | GBSS1-R1 | GGGCTGGAGAAATCAACAAG |
| (*Waxy*) | GBSS1-2 | GBSS1-F2 | CCCCTCTCTCACCATTCCTT | GBSS1-R2 | AGTTTCTTGGGTGGCTAGGG |
|  | GBSS1-3 | GBSS1-F3 | GCATGAACGTCGTGTTCGTC | GBSS1-R3 | TCTTCAGGTAGCTCGCCAGT |
|  | GBSS1-4 | GBSS1-F4 | GAGAAGTATCCGGGCAAGGT | GBSS1-R4 | CACATGTTTGACCGTTCGTC |
|  | GBSS1-5 | GBSS1-F5 | TGGGTTCGCTTCTCTTCTCT | GBSS1-R5 | TTTCCAGCCCAACACCTTAC |
| *SSS1* | SSS1-1 | SSS1-F1 | CACTGAATTGTGGTCGATGG | SSS1-R1 | GTTGTGTCATGGGTGATGGT |
|  | SSS1-2 | SSS1-F2 | CTGTTTGGCTTTTCCATTGC | SSS1-R2 | GGAACATGCCAAAACAAAGG |
|  | SSS1-3 | SSS1-F3 | GAGAGTGCGTGGTACATGCT | SSS1-R3 | GCGTTCACAATGAAAGCAATAG |
|  | SSS1-4 | SSS1-F4 | GCAGTTGTGACAGCAGATCG | SSS1-R4 | CTTGTCTGTGGATGGGTTCC |
|  | SSS1-5 | SSS1-F5 | GCGGGACAATATTCAATTCG | SSS1-R5 | CAAGGTTCGAATCTGGATGG |
|  | SSS1-6 | SSS1-F6 | GTTTCCCACCGAATAACTGC | SSS1-R6 | GTGTAATGCTTGTGCCCTGA |
|  | SSS1-7 | SSS1-F7 | GGCCCATTTTGTGTCATTG | SSS1-R7 | TTATATTGCGCGACGGACT |
| *SSS2A* | SSS2A-1 | SSS2A-F1 | CCTTCCATCCGTAGACAAGC | SSS2A-R1 | GCGTAATCACCGTACCTTGG |
|  | SSS2A-2 | SSS2A-F2 | AGATCGGAAACGGGGACTCT | SSS2A-R2 | GAGTTTATCGTGTCCCTGCTC |
