# Supplementary material for: Molecular Aspect of Good Eating Quality Formation in Japonica Rice
Source: PLoS One. 2011 Apr 6;6(4):e18385. doi: 10.1371/journal.pone.0018385 (PMC3071818; doi:10.1371/journal.pone.0018385)
Supplement: Table S2 — Primers for SBE1 and SBE3 expressions in developing rice grains. (DOC) [file pone.0018385.s002.doc]

**Table S2.** Primers for *SBE1* and *SBE3* expressions in developing rice grains.

| **Enzyme** | **Gene** | **EC no.** | **Acc. no.** | **Chr.** | **Primer sequences** | **Product size (bp)** |
| --- | --- | --- | --- | --- | --- | --- |
| Starch branching enzyme 1 | *SBE1* | 2.4.1.18 | AK068920 | 6 | F: CTACCATCAACCGTGGCATT | 182 |
|  |  |  |  |  | R: GTCGACAAGGCTCCACTGAC |  |
| Starch branching enzyme 3 | *SBE3* | 2.4.1.18 | EU735074 | 2 | F: TGCTCATGGATGTTGTTCACAG | 200 |
|  |  |  |  |  | R: ATACTCCTCGAGCCACCATCTT |  |
