# Supplementary material for: Molecular Aspect of Good Eating Quality Formation in Japonica Rice
Source: PLoS One. 2011 Apr 6;6(4):e18385. doi: 10.1371/journal.pone.0018385 (PMC3071818; doi:10.1371/journal.pone.0018385)
Supplement: Table S3 — Eating quality parameters for eight japonica rice varieties. (DOC) [file pone.0018385.s003.doc]

**Table S3.** Eating quality parameters for eight *japonica* rice varieties.

| **Variety** | **P** ^a^ | **AAC(%)** | **PC(%)** | **ADV** | **PV** ^b^ | **HPV** ^b^ | **CPV** ^b^ | **BDV** ^b^ | **SBV** ^b^ | **CTV** ^b^ | **PT(℃)** |
| --- | --- | --- | --- | --- | --- | --- | --- | --- | --- | --- | --- |
| Gopum | 70.4 a ^c^ | 18.00 ab | 6.13 f | 6.3 cd | 276.07 b | 163.45 c | 269.90 b | 112.62 c | -6.17 c | 106.44 cd | 68.08 cd |
| Koshihikari | 68.0 a | 17.59 b | 6.33 f | 6.3 bc | 317.56 a | 203.68 a | 307.57 a | 113.88 c | -9.98 c | 103.89 d | 68.03 de |
| Ilpum | 63.6 b | 18.67 a | 6.63 e | 6.5 a | 261.34 c | 185.45 b | 295.64 a | 75.90 e | 34.29 a | 110.19 bc | 68.15 b |
| Samgwang | 63.5 b | 18.08 ab | 5.78 g | 6.5 a | 284.69 b | 158.79 c | 273.75 b | 125.90 b | -10.94 c | 114.96 ab | 68.13 bc |
| Palgong | 60.4 b | 17.71 b | 7.10 d | 6.3 bcd | 286.31 b | 185.19 b | 294.70 a | 101.12 d | 8.39 b | 109.52 c | 68.00 e |
| Samnam | 55.9 c | 17.57 b | 7.95 c | 6.2 d | 279.19 b | 168.03 c | 271.29 b | 111.16c | -7.90 c | 103.26 d | 68.05 de |
| Singeumo | 51.9 cd | 17.74 b | 8.23 b | 6.4 b | 241.45 d | 165.85 c | 281.16 b | 75.60 e | 39.71 a | 115.31 a | 68.03 de |
| Dobong | 49.7 d | 14.35 c | 8.54 a | 1.5 e | 314.97 a | 166.32 c | 247.37 c | 148.65 a | -67.60 d | 81.05 e | 74.28 a |

^a^ P, palatability value; AAC, apparent amylose content; PC, protein content; ADV, alkali digestion value; PV, peaking value; HPV, hot pate viscosity; CPV, cool paste viscosity; BDV, breakdown value; SBV, setback viscosity; CTV, consistency viscosity; PT, pasting temperature (℃);^b^ RVU: rapid visco unit; ^c^ Letters a-g, difference is significant at 0.05 level.
