# Supplementary material for: Molecular Aspect of Good Eating Quality Formation in Japonica Rice
Source: PLoS One. 2011 Apr 6;6(4):e18385. doi: 10.1371/journal.pone.0018385 (PMC3071818; doi:10.1371/journal.pone.0018385)
Supplement: Table S4 — Polymorphic sites of genes related to rice amylopectin biosynthesis of eight japonica varieties. (DOC) [file pone.0018385.s004.doc]

**Table S4.** Polymorphic sites of genes related to rice amylopectin biosynthesis of eight *japonica* varieties.

|  | **Variety** | | | | | | | |  |  |
| --- | --- | --- | --- | --- | --- | --- | --- | --- | --- | --- |
| **SNP** | Gopum | Koshihikari | Ilpum | Samgwang | Palgong | Samnam | Singeumo | Dobong | **Location** | **Code** |
| *SBE1*_30897557 **^a^** | T **^b^** | C | C | C | C | C | C | C | Exon 2 | S1 |
| *SBE1*_30897629 | T | T | T | C | T | C | T | C | Exon 2 | S2 |
| *SBE1*_30897815 | T | T | T | C | T | T | T | C | Exon 2 | S3 |
| *SBE1*_30897858 | T | T | T | C | T | T | T | C | Exon 2 | S4 |
| *SBE1*_30897972 | T | C | T | C | T | T | T | C | Exon 2 | S5 |
| *SBE1*_30898299 | A | A | A | A | G | G | G | A | Intron 2 | S6 |
| *SBE1*_30898310 | G | A | G | A | A | G | A | G | Intron 2 | S7 |
| *SBE1*_30898319 | G | G | G | G | G | A | A | G | Intron 2 | S8 |
| *SBE1*_30898321 | G | G | G | A | A | G | G | G | Intron 2 | S9 |
| *SBE1*_30898341 | G | A | A | A | A | A | A | A | Intron 2 | S10 |
| *SBE1*_30898447 | A | A | A | G | G | G | G | A | Intron 2 | S11 |
| *SBE1*_30898512 | G | G | G | G | G | G | G | A | Intron 2 | S12 |
| *SBE1*_30898530 | G | G | G | G | G | G | G | A | Intron 2 | S13 |
| *SBE1*_30898565 | G | A | G | G | G | A | G | A | Intron 2 | S14 |
| *SBE1*_30899579 | G | T | T | T | T | T | T | T | Intron 4 | S15 |
| *SBE1*_30900357 | T | T | T | T | T | T | C | T | Exon 6 | S16 |
| *SBE1*_30900359 | C | G | A | C | C | C | C | C | Exon 6 | S17 |
| *SBE1*_30902277 | G | G | G | G | G | G | A | G | Exon 9 | S18 |
| *SBE1*_30902314-15 | - **^c^** | - | - | - | - | - | T | - | Intron 9 | S19 |
| *SBE1*_30902332-33 | - | - | - | T | - | T | - | - | Intron 9 | S20 |
| *SBE3*_19351418 | G | A | A | A | A | A | T | A | Intron 3 | S21 |
| *SBE3*_19352538-39 | - | G | G | - | G | G | - | - | Intron 6 | S22 |
| *SBE3*_19353180 | C | C | C | A | C | C | C | C | Intron 8 | S23 |
| *SBE3*_19353183 | C | A | A | A | C | A | C | C | Intron 8 | S24 |
| *SBE3*_19354868 | A | A | C | A | A | A | A | A | Intron 11 | S25 |
| *SBE3*_19356138 | T | T | T | C | T | T | T | T | Exon 14 | S26 |
| *SBE3*_19356187 | G | G | G | T | G | G | G | G | Intron 14 | S27 |
| *SBE3*_19356196 | T | T | T | G | T | T | T | T | Intron 14 | S28 |
| *SBE3*_19356275 | T | T | T | - | T | T | T | T | Intron 14 | S29 |
| *SBE3*_19356280-81 | - | - | - | - | - | T | - | - | Intron 14 | S30 |
| *SBE3*_19357788 | T | A | T | T | T | A | T | T | Intron 15 | S31 |
| *SBE3*_19357791 | A | A | A | A | A | T | A | A | Intron 15 | S32 |
| *SBE3*_19357793 | A | C | A | A | A | C | A | A | Intron 15 | S33 |
| *SBE3*_19357801 | A | A | A | A | A | G | A | A | Intron 15 | S34 |
| *SBE3*_19357805 | A | T | A | A | A | T | A | A | Intron 15 | S35 |
| *SBE3*_19357861 | A | T | A | A | A | A | A | A | Intron 15 | S36 |
| *SBE3*_19357899 | G | G | A | G | G | G | A | A | Intron 15 | S37 |
| *SBE3*_19357949 | T | T | T | T | T | A | T | T | Intron 15 | S38 |
| *SBE3*_19357977 | C | C | C | C | C | T | C | C | Intron 15 | S39 |
| *SBE3*_19357988 | G | C | G | G | G | G | G | C | Intron 15 | S40 |
| *SBE3*_19357996 | C | G | G | G | C | G | G | G | Intron 15 | S41 |
| *SBE3*_19358019 | G | A | A | A | A | A | A | A | Intron 15 | S42 |
| *SBE3*_19358823 | - | G | G | G | G | G | G | G | Exon 17 | S43 |
| *SBE3*_19358829 | T | C | C | C | C | C | G | C | Exon 17 | S44 |
| *SBE3*_19360797 | C | C | C | - | C | C | C | C | Intron 21 | S45 |
| *SBE3*_19360867 | C | C | C | A | C | C | C | C | Exon 22 | S46 |
| *GBSS1*_1764506 | T | G | G | G | G | G | G | G | 5' UTR | S47 |
| *GBSS1*_1764705 | C | - | C | C | C | C | C | - | Exon 1 | S48 |
| *GBSS1*_1764706 | T | - | T | T | T | T | T | - | Exon 1 | S49 |
| *GBSS1*_1764795-96 | - | - | C | - | - | - | - | - | Intron 1 | S50 |
| *GBSS1*_1768059 | T | C | C | C | C | T | C | T | Exon 10 | S51 |
| *SSS1*_3079872-73 | - | T | - | - | - | T | T | T | Intron 2 | S52 |
| *SSS1*_3080102 | C | A | C | A | A | A | C | A | Intron 2 | S53 |
| *SSS1*_3082037 | T | T | - | - | T | - | T | - | Intron 6 | S54 |
| *SSS1*_3082239-40 | - | - | - | - | - | - | - | T | Intron 7 | S55 |
| *SSS1*_3085680-81 | - | - | A | - | - | - | - | - | Exon 15 | S56 |
| *SSS1*_3085740-41 | - | - | C | - | - | - | - | - | Exon 15 | S57 |
| *SSS2A*_6749092 | A | A | A | A | A | A | A | - | Intron 3 | S58 |
| *SSS2A*_6749540 | - | A | - | - | - | - | - | - | Intron 3 | S59 |
| *SSS2A*_6749628 | T | T | T | T | G | T | T | T | Intron 4 | S60 |

^a^ *SBE1,* LOC_Os06g51084, MSU6: 6:30895778:30905417:-1; *SBE3,* LOC_Os02g32660, MSU6: 2:19349280:19361859:-1; *GBSS1,* LOC_Os06g04200, MSU6: 6:1764023:1770257:1; *SSS1,* LOC_Os06g06560, MSU6: 6:3077460:3086409:-1; *SSS2A,* LOC_Os06g12450, MSU6: 6:6746759:6752939:1; ^b^ AGTC, DNA bases; - ^c^, insertion-deletion.
